# Synthesis and Characterization of (*Z*)-5-Arylmethylidene-rhodanines with Photosynthesis-Inhibiting Properties ^†^

**DOI:** 10.3390/molecules16065207

**Published:** 2011-06-22

**Authors:** Veronika Opletalova, Jan Dolezel, Katarina Kralova, Matus Pesko, Jiri Kunes, Josef Jampilek

**Affiliations:** 1 Department of Pharmaceutical Chemistry and Drug Control, Faculty of Pharmacy in Hradec Kralove, Charles University in Prague, Heyrovskeho 1203, 500 05 Hradec Kralove, Czech Republic; 2 Institute of Chemistry, Faculty of Natural Sciences, Comenius University, Mlynska dolina Ch-2, 842 15 Bratislava, Slovakia; 3 Department of Ecosozology and Physiotactics, Faculty of Natural Sciences, Comenius University, Mlynska dolina Ch-2, 84215 Bratislava, Slovakia; 4 Department of Inorganic and Organic Chemistry, Faculty of Pharmacy in Hradec Kralove, Charles University in Prague, Heyrovskeho 1203, 500 05 Hradec Kralove, Czech Republic; 5 Department of Chemical Drugs, Faculty of Pharmacy, University of Veterinary and Pharmaceutical Sciences, Palackeho 1/3, 612 42 Brno, Czech Republic

**Keywords:** rhodanine derivatives, synthesis, lipophilicity, photosynthesis inhibition, spinach chloroplasts, *Chlorella vulgaris*, structure-activity relationships

## Abstract

A series of rhodanine derivatives was prepared. The synthetic approach, analytical and spectroscopic data of all synthesized compounds are presented. Lipophilicity of all the discussed rhodanine derivatives was analyzed using the RP-HPLC method. The compounds were tested for their ability to inhibit photosynthetic electron transport (PET) in spinach (*Spinacia oleracea* L.) chloroplasts and reduce chlorophyll content in freshwater alga *Chlorella vulgaris*. Structure-activity relationships between the chemical structure, physical properties and biological activities of the evaluated compounds are discussed. For majority of the tested compounds the lipophilicity of the compound and not electronic properties of the R^1^ substituent were decisive for PET-inhibiting activity. The most potent PET inhibitor was (5*Z*)-5-(4-bromobenzylidene)-2-thioxo-1,3-thiazolidin-4-one (IC_50_ = 3.0 μmol/L) and the highest antialgal activity was exhibited by (5*Z*)-5-(4-chlorobenzylidene)-2-thioxo-1,3-thiazolidin-4-one (IC_50_ = 1.3 μmol/L).

## 1. Introduction

Pesticides are used to control pests. About 700 pesticides, including insecticides, herbicides and fungicides, act on perhaps some 95 biochemical targets in insects, weeds and destructive fungi. They must be effective without human or crop injury and safe relative to humans and the environment. Herbicides act mostly in plant-specific pathways, e.g., by blocking photosynthesis. Green plant pigments absorb light and with the coupled system of chloroplasts convert light energy to the chemical energy of adenosine triphosphate. Herbicides disrupting some of the processes unique to plants are of low toxicity to mammals, which lack analogous targets. Photosystem (PS) II was an early target for herbicides and is still highly important as the mode of action for about 50 commercial compounds. More than one target is involved since resistance to one PS II inhibitor does not confer cross-resistance to all others. The targets are denoted as the triazine, urea, and nitrile sites [1].

PS II electron transport inhibitors bind to the D1 protein of the PS II reaction centre, thus blocking electron transfer to plastoquinone. The inhibition of PS II electron transport prevents the conversion of absorbed light energy into electrochemical energy and results in production of triplet chlorophyll and singlet oxygen, which induces the peroxidation of membrane lipids [2]. The interaction of herbicides with the photosynthetic apparatus and a model for orientation of the herbicides within the three-dimensional structure of their target, the D1 protein of PS II, were reported by Draber *et al.* [3]. Many QSAR studies of PS II inhibitors with diverse chemical structures have emphasized the hydrophobic nature of the binding domain, with lipophilicity being the dominant determinant of Hill inhibition activity [4].

Rhodanine represents an important scaffold in drug discovery [5], and the influence of its derivatives on plant physiology has been well documented, too [6,7,8,9,10,11,12,13,14,15,16]. 5-Arylalkylidenerhodanines [7] and 3-arylrhodanines [9] were patented as potential herbicides, and 5-(5-barbiturilidene)rhodanine inhibited growth of algae in water at relatively low concentrations [8]. Herbicidal activity of complexes of transition metals with rhodanine [12] was reported as well. Rhodanine derivatives also inhibit diaminopimelate aminotransferase, an enzyme catalyzing L-lysine synthesis in plants and bacteria but not in mammals that acquire this essential amino acid in their diet. Specific inhibitors of this enzyme could thus potentially serve as herbicides and antibiotics that are non-toxic to mammals [16].

The inhibition of photosynthetic electron transport by rhodanine (IC_50_ ≈ 1 mmol/L) was observed by Muro *et al.* [14]. In another study, rhodanine and rhodanine-*N*-acetic acid strongly inhibited growth of *Daucus carota* L. var. *sativa* DC even at low concentration 0.3 mmol/L. Both compounds also inhibited germination of seeds of *Daucus carota* L. var. *sativa* DC and *Sesamum indicum* (at 1 mmol/L) [13]. Chlorophyll synthesis in the cotyledons of *Brassica rapa* L. was strongly inhibited with rhodanine at the concentration of 0.3 mmol/L [11]. Similar results were later observed with *N*–aminorhodanine [13,15]. It was found that the free amino group at *N–*3 was essential for the greater inhibitory activity of rhodanine derivatives. It was also confirmed that the plant-growth inhibition by these derivatives was related to the chlorophyll content in treated plants [13].

Many low molecular weight drugs cross biological membranes through passive transport, which strongly depends on their lipophilicity. This property has a major effect on absorption, distribution, metabolism, excretion and toxicity (ADME/Tox) properties as well as biological activity. Lipophilicity has been studied and applied as an important drug property for decades [17]. This paper is a follow-up work to previous papers [18,19,20,21,22,23] aimed at studying relationships between the structure and lipophilicity of various compounds and their biological effects.

## 2. Results and Discussion

### 2.1. Chemistry

**Scheme 1:**
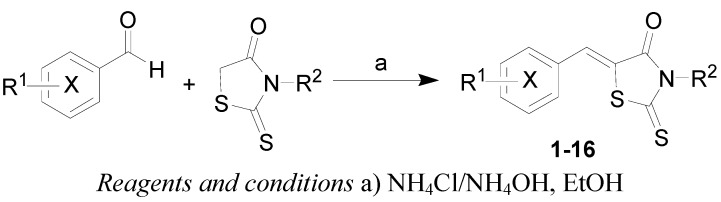
Synthesis of target rhodanine derivatives **1**–**16**.

The synthesis of compounds is indicated in Scheme 1, and the compounds are listed in Table 1. Either commercially available rhodanines or prepared 3-(2-hydroxyethyl)rhodanine [24] and pyrazine-2–carbaldehyde [25] were used as starting materials. Most of the compounds were reported previously [26,27,28,29,30,31,32,33,34]. (5*Z*)-3-(2-Hydroxyethyl)-5-(2-nitrobenzylidene)-2-thioxo-1,3-thiazolidin-4-one (**11a**), (5*Z*)-3-(2-hydroxyethyl)-5-(3-nitrobenzylidene)-2-thioxo-1,3-thiazolidin-4-one (**11b**) and (5*Z*)-5-(pyrazin-2-ylmethylidene)-2-thioxo-1,3-thiazolidin-4-one (**15**) are novel compounds.

Arylmethylidenerhodanines can form two isomers. According to references [35,36,37,38,39], syntheses of these compounds results in the *Z*–izomer. Configuration on the exocyclic double bond can be determined on the basis of NMR spectra where ^1^H-NMR signals of the methine-group hydrogens for *Z–*isomers are more downfield compared to those of the *E*–isomers The experimental signals of methine-group hydrogens in the rhodanine derivatives studied in the present paper were compared with the values reported previously and the values predicted *in silico* (Table 1). It can be concluded that all arylmethylidenerhodanines reported in the present paper were obtained as single (*Z*)–isomers. In most cases experimental values are between the values predicted with CS ChemOffice 7.0 and those predicted with CS ChemOffice 10.0.

### 2.2. Lipophilicity

Hydrophobicities (log *P*/Clog *P*) of compounds **1**–**16** were calculated using two commercially available programs (ChemDraw Ultra 10.0 and ACD/LogP), and also measured by means of RP-HPLC determination of capacity factors *k* with a subsequent calculation of log *k*. The procedure was performed under isocratic conditions with methanol as an organic modifier in the mobile phase using an end-capped non-polar C_18_ stationary RP column. The ChemDraw program did not resolve various lipophilicity values of individual positional isomers, that is, the same log *P*/Clog *P* data were calculated for isomers **a**–**c**, respectively for positional isomers **13** and **14**. Due to high functionalization of these small molecules, the program ACD did not resolve various lipophilicity values of individual positional isomers **7**–**11** as well as **12** and **13**. Therefore it can be assumed that the determined log *k* data specify lipophilicity within the individual series of compounds. The results are summarized in Table 1.

**Table 1:** Comparison of ^1^H-NMR signals of methine-group for *Z*-isomers and comparison of calculated lipophilicities (log *P*, Clog *P*) with determined log *k* values of compounds. 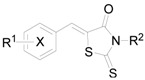

**Comp.**	X	R^1^	R^2^	Predicted values	Exp. values	Exp. values reported previously	log *k*	log *P*^*b*^Clog *P*^*b*^	log *P*^*j*^
**1**	C	H	H	7.42 *^a^*,7.80*^b^*	7.63	7.63 *^c^*,7.65*^d^*, 7.62*^g^*	0.5122	2.041.802	2.94± 0.76
**2a**	C	2-OH	H	7.69 *^a^*, 8.07*^b^*	7.84	7.83 *^c^*, 7.86*^e^*	0.4664	1.651.135	2.21± 0.76
**2b**	C	3-OH	H	7.42 *^a^*,7.80*^b^*	7.53	7.54 *^e^*	0.2744	1.651.135	2.86± 0.77
**2c**	C	4-OH	H	7.42 *^a^*,7.80*^b^*	7.55	7.56 *^e^*, 7.56*^g^*	0.2641	1.651.135	2.96± 0.77
**3**	C	2,4-OH	H	7.69 *^a^*, 8.07*^b^*	7.73	7.79 *^f^*	0.2776	1.260.468	2.23± 0.78
**4a**	C	2-OCH_3_	H	7.69 *^a^*, 8.07*^b^*	7.78	7.79 *^e^*	0.5867	1.911.721	2.95± 0.77
**4b**	C	3-OCH_3_	H	7.42 *^a^*, 7.80*^b^*	7.60	7.59 *^e^*	0.5713	1.911.721	2.92± 0.77
**4c**	C	4-OCH_3_	H	7.42 *^a^*, 7.80*^b^*	7.59	7.59 *^c^*,7.52*^d^*, 7.45*^e^*,	0.5425	1.911.721	2.89± 0.77
**5**	C	3-OCH_3_-4-OH	H	7.42 *^a^*, 7.80*^b^*	7.56	7.94 *^g^*	0.3553	1.520.984	2.72± 0.78
**6**	C	4-N(CH_3_)_2_	H	7.42 *^a^*, 7.80*^b^*	7.49	7.47 *^c^*	0.6466	2.321.967	3.05± 0.77
**7a**	C	2-NO_2_	H	7.98 *^a^*, 8.36*^b^*	7.86	7.82 *^c^*	0.2254	2.491.545	2.67± 0.77
**7b**	C	3-NO_2_	H	7.53 *^a^*, 7.91*^b^*	7.70	7.79 *^h^*	0.2399	2.491.545	2.67± 0.77
**7c**	C	4-NO_2_	H	7.56 *^a^*, 7.94*^b^*	7.70	7.73 *^h^*	0.2436	2.491.545	2.67± 0.77
**8a**	C	2-F	H	7.69 *^a^*, 8.07*^b^*	7.59	7.59 *^d^*, 7.48*^e^*	0.8108	2.191.945	2.99± 0.81
**8b**	C	3-F	H	7.42 *^a^*,7.80*^b^*	7.63	7.83 *^e^*	0.8204	2.191.945	2.99± 0.81
**8c**	C	4-F	H	7.42 *^a^*, 7.80*^b^*	7.64	7.65 *^d^*	0.7909	2.191.945	2.99± 0.81
**9a**	C	2-Cl	H	7.69 *^a^*, 8.07*^b^*	7.74	NR	0.9019	2.592.515	3.54± 0.77
**9b**	C	3-Cl	H	7.42 *^a^*, 7.80*^b^*	7.68	NR	1.0270	2.592.515	3.54± 0.77
**9c**	C	4-Cl	H	7.42 *^a^*_, _7.80*^b^*	7.62	7.61 *^c^*,7.55*^d^*, 7.61*^i^*	0.5936	2.592.515	3.54± 0.77
**10a**	C	2-Br	H	7.69 *^a^*, 8.07*^b^*	7.70	NR	0.9368	2.862.665	3.91± 0.81
**10b**	C	3-Br	H	7.42 *^a^*, 7.80*^b^*	7.61	NR	1.0820	2.862.665	3.71± 0.81
**10c**	C	4-Br	H	7.42 *^a^*, 7.80*^b^*	7.60	7.61 *^d^*	0.6940	2.862.665	3.71± 0.81
**11a**	C	2-NO_2_	C_2_H_4_OH	7.98 *^a^*, 8.36*^b^*	7.88	NR	0.4751	2.040.993	1.81± 0.80
**11b**	C	3-NO_2_	C_2_H_4_OH	7.53 *^a^*, 7.91*^b^*	7.94	NR	0.6077	2.040.993	1.81± 0.80
**11c**	C	4-NO_2_	C_2_H_4_OH	7.56 *^a^*, 7.94*^b^*	7.88	NR	0.6349	2.040.993	1.81± 0.80
**12**	2-N	H	H	7.63 *^a^*, 7.63*^b^*	7.67	7.65 *^d^*	0.4864	1.120.305	1.45± 0.76
**13**	3-N	H	H	7.42 *^a^*, 7,68*^b^*	7.66	7.60 *^d^*	0.4539	0.700.305	1.70± 0.77
**14**	4-N	H	H	7.40 *^a^*, 7.78*^b^*	7.55	7.58 *^d^*	0.1878	0.700.305	1.45± 0.76
**15**	2,4-N	H	H	7.42 *^a^*, 7.42*^b^*	7.73	NR	0.2359	-0.22-0.652	0.69± 0.77
**16**	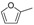	H	H	7.42 *^a^*, 7.37*^b^*	7.47	8.09 *^g^*	0.4656	0.650.978	2.10± 0.77

*^a^* CS ChemOffice 7.0, *^b^* CS ChemOffice 10.0 (CambridgeSoft, Cambridge, MA, U.S.A.); *^c^* ref. [16]; *^d^* ref. [26]; *^e^* ref. [27]; *^f^* ref. [28]; *^g^* ref. [35]; *^h^* ref. [38]; *^i^* ref. [39]; NR = not reported; *^j^* ACD/LogP 1.0 (Advanced Chemistry Development, Toronto, Canada).

Compounds **14** (pyridin-4-ylmethylidene) and **7a** (2-nitrobenzylidene) showed the lowest lipophilicity, while compound **10b** (3-bromobenzylidene) exhibited the highest. Generally, it can be concluded that ring-substituted 5-benzylidene derivatives are more lipophilic than their 5-heteroarylmethylidene congeners. Unsubstituted (5*Z*)-5-benzylidene-2-thioxo-1,3-thiazolidin-4-one (**1**) is situated approx. in the middle of the lipophilicity range of the compound series.

When the lipophilicity of 5-heteroarylmethylidene-2-thioxo-1,3-thiazolidin-4-ones **12**–**16** and compound **1** was compared, the lowest lipophilicity was surprisingly shown by compound **14** (pyridin-4-ylmethylidene) contrary to expected compound **15** (pyrazin-2-ylmethylidene), as it was predicted by all calculated log *P*/Clog *P* data. Lipophilicity within 5-heteroarylmethylidene series increased in the following order: pyridin-4-yl (**14**) < pyrazin-2-yl (**15**) < pyridin-3-yl (**13**) < furan-2-yl (**16**) < pyridin-2-yl (**12**); it is lower than the lipophilicity of unsubstituted benzylidene (**1**).

In 5-benzylidene series **1**–**11** nitro (**7a**–**c**) and hydroxyl (**2a**–**c**) substituted compounds possessed lower lipophilicity than unsubstituted compound **1**. Compound **2a** (2-OH) showed higher experimental lipophilicity value in comparison with the results calculated by the software and in comparison with other two isomers **2b** (3-OH) and **2c** (4-OH). Surprisingly, disubstituted derivatives **11a**–**c** showed dramatically higher lipophilicity than monosubstituted nitro derivatives **7a**–**c**. Lipophilicity in both cases increased in the order: 2-NO_2_ (**a**) < 3-NO_2_ (**b**) < 4-NO_2_ (**c**). Methoxy (**4a**–**c**) and halogeno (**8**–**10**) derivatives were more lipophilic than unsubstituted parent compound **1**. Experimental lipophilicity values of methoxy derivatives **4a**–**c** were higher than it was expected on the basis of log *P* values calculated *in silico* and similarly as those of hydroxyl derivatives **2a**–**c** increased in the following order: 4-OCH_3_/4-OH (**c**) < 3-OCH_3_/3-OH (**b**) < 2-OCH_3_/2-OH (**a**). Generally, fluoro derivatives **8a**–**c** showed lower lipophilicity than chloro derivatives **9a**–**c**, and both showed lower lipophilicity than bromo derivatives **10a**–**c**. Depending on the substituent position, lipophilicity within substituted 5-benzylidene series increased in the following order: 4- (**c**) < 2- (**a**) < 3- (**b**).

In general, experimentally-determined log *k* values correlated relatively poorly with the calculated log *P*/Clog *P*. These facts are possibly caused by limitations of the software used and intramolecular interactions between heteroatoms and substituents. Based on the facts discussed above, it can be stated that the lipophilicity of the discussed compounds is significantly influenced by intramolecular interactions.

### 2.3. Study of PET Inhibition in Spinach Chloroplasts

The inhibitory activity (IC_50_ values) of rhodanine derivatives related to inhibition of photosynthetic electron transport (PET) in spinach (*Spinacia oleracea* L.) chloroplasts is summarized in Table 2. IC_50_ values of compounds **2c**, **3**, **4a**, **5**, **6**, **11c**, **12** and **14** could not be determined due to low solubility of the compounds in the chloroplast suspension or due to very weak activity of the compounds; compound **11b** interacted with the artificial electron acceptor 2,6-dichlorophenol-indophenol.

Replacement of H by C_2_H_4_OH group in R^2^ substituent (compounds **7a** and **11a**, respectively) causing the increase of log *k* from 0.2254 to 0.4751 led to a slight activity increase. Similarly, more lipophilic compound **2a** with R^1^: 2-OH (log *k* = 0.4664) exhibited higher inhibitory activity than 4-OH substituted compound **2c** (log *k* = 0.2641), see Table 1 and Table 2.

**Figure 1:**
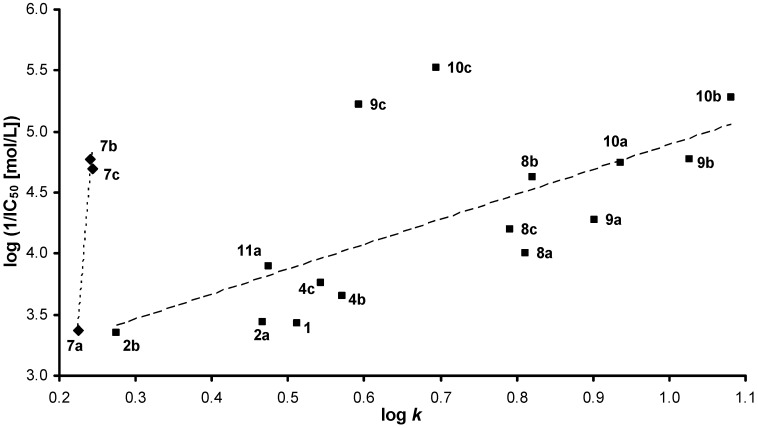
Dependence of log (1/IC_50_ [mol/L]) related to PET inhibition in spinach chloroplasts on the compound lipophilicity expressed by log *k*.

The dependence of inhibitory activity on compound lipophilicity expressed by log *k* for compounds with X=C (benzylidene derivatives) is shown in Figure 1. It is evident that particularly high inhibitory activity was exhibited by halogen substituted compounds **10c** (4-Br) and **9c** (4-Cl), and also with **7b** (3-NO_2_). High PET-inhibiting potency was also observed for **10b** (3-Br) and **9b** (3-Cl), and for **7c** (4-NO_2_). Hence, electron-withdrawing substituents with higher values of Hammett's constants (σ constants for 4-Br: 0.232, 3-Br: 0.390, 4-Cl: 0.227, 4-NO_2_: 1.238, 3-NO_2_: 0.710 [40]) seem to contribute to the PET-inhibitory activity which is in a good agreement with the results obtained in the experiment with *Chlorella vulgaris*, see below. The superior activities of **9c** and **10c** in comparison with those of other compounds with similar lipophilicity could be connected with the favourable steric and electronic properties of 4-halogeno substituted compounds with respect to the site of inhibitory action in PS II of spinach chloroplasts.

**Table 2:** The inhibitory activity of the selected rhodanine derivatives related to the inhibition of photosynthetic electron transport (PET inhibition) in spinach chloroplasts (*Spinacia oleracea* L.) as well as their activity related to the reduction of chlorophyll content in *Chlorella vulgaris* (expressed as IC_50_ values or as reduction of chlorophyll content [%] caused by application of 100 μmol/L of the studied compound) in comparison with standard 3-(3,4-dichlorophenyl)-1,1-dimethylurea DCMU.

**Comp.**	Spinach chloroplasts (PET) IC_50_ [μmol/L]	*Chlorella* *vulgaris*	
		IC_50_ [μmol/L]	reduction of Chl. cont. [%]
**1**	374.7	13.7	88.2
**2a**	368.6	59.4	59.0
**2b**	444.0	–	9.6
**2c**	*^a^*	–	29.8
**3**	*^a^*	108.2	48.5
**4b**	220.6	*^a^*	–
**4c**	173.8	*^a^*	–
**5**	*^a^*	–	19.4
**6**	*^a^*	–	12.6
**7a**	427.6	*^a^*	–
**7b**	16.9	4.4	85.7
**7c**	20.1	21.9	87.1
**8a**	99.5	*^a^*	–
**8b**	23.8	*^a^*	–
**8c**	63.5	*^a^*	–
**9a**	53.3	*^a^*	–
**9b**	17.0	*^a^*	–
**9c**	6.0	1.3	84.8
**10a**	18.1	*^a^*	–
**10b**	5.2	*^a^*	–
**10c**	3.0	*^a^*	–
**11a**	127.4	*^a^*	–
**13**	310.7	*^a^*	–
**15**	216.5	*^a^*	1.8
**DCMU**	1.9	7.3	–

*^a^* interaction with DCPIP or precipitation during the experiment.

Halogen as well as nitro substituents contributed to enhanced PET-inhibiting activity of 2,6-disubstituted 4-amidopyridines and 4-thioamidopyridines [41], pyrazine-2-carboxanilides [42,43], derivatives of 3-nitro-2,4,6-trihydroxybenzamide [44], substituted benzanilides and thiobenzanilides [45,46,47], or antialgal/PET-inhibiting activity of quinoline derivatives [47,48,49,50,51,52] and substituted salicylanilides [53]. Nonetheless, it is evident from the results of statistical analysis that the inhibitory activity of 12 compounds with X=C for which IC_50_ in mol/L could be determined depended predominantly on compound lipophilicity expressed as log *k*, see Figure 1.

log (1/IC_50_) = 2.411 (± 0.211) + 2.356 (± 0.276) log *k*

r = 0.938, s = 0.229, F = 73.0, n = 12

The IC_50_ value of the unsubstituted rhodanine related to PET inhibition in spinach chloroplasts was previously determined by Muro *et al.* [14] using spinach chloroplasts. However, the IC_50_ value of approximately 1 mmol/L which was obtained for rhodanine during the normal 1 min assay decreased to 0.1 mmol/L when the assay was done after illumination for 3 min, indicating possible chemical modification of the compound.

### 2.4. Reduction of Chlorophyll Content in *Chlorella vulgaris*

The inhibitory activities (IC_50_ values) of rhodanine derivatives related to the reduction of chlorophyll content in *Chlorella vulgaris* algae are summarized in Table 2. Ten compounds were tested for their inhibitory potency to reduce chlorophyll content in *C. vulgaris* suspension. Only for six of them (**1**, **2a**, **3**, **7b**, **7c**, **9c**) the IC_50_ value, *i.e.*, concentration causing 50% reduction of chlorophyll concentration, could be determined, see Table 2. Therefore, the extent of chlorophyll content reduction in *C. vulgaris* suspension treated with equimolar concentration (100 μmol/L) of the studied compounds (**1**-**3**, **5**, **6**, **7b**, **7c**, **9c**) was compared as well, see Table 2.

Based on dependencies of IC_50_ on the lipophilicity expressed by log *k*, the compounds could be divided into 2 groups. For compounds with lower values of log *k*, ranging from 0.2399 (**7b**) to 0.2776 (**3**), the inhibitory activity decreased linearly with increasing log *k* value, whereas for compounds with log *k* raging from 0.4664 (**2a**) to 0.5936 (**9c**) the reverse relationship was observed.

Similar results were obtained also for the dependence of the reduction of chlorophyll content in *C. vulgaris* suspension treated with equimolar concentration (100 μmol/L) of the compounds on the log *k* values of the compounds. However, for the most lipophilic compound in the set (**6**, log *k*: 0.6466) strong decrease in potency was observed (Table 2). Thus, it could be assumed that in the investigated set of compounds higher values of Hammett's σ constants of the R^1^ (4-Cl: 0.227, 4-NO_2_: 1.238, 3-NO_2_: 0.710 [40]) substituent contributed significantly to the increase of biological activity. Muro *et al.* [14] found that rhodanine applied at 1 mmol/L concentration completely inhibited growth of immature cells of *Marchantia polymorpha* within 90 hours and caused decrease of chlorophyll content. Algicidal properties of 5-(5-barbiturilidene)rhodanine against the algae species *Scenedesmus, Plectonema, Anabena, Ankistrodesmus, Oscillatoria, Coccochloris, Chlamydomonas, Lyngbya, Synura* and *Chlorella* were reported by Kerst *et al.* [8].

## 3. Experimental

### 3.1. General

Commercially available rhodanine and aldehydes were used as starting materials. Methods reported previously were employed for preparation of 3-(2-hydroxyethyl)rhodanine [24] and pyrazine-2–carbaldehyde [25]. For analysis, the samples of compounds were dried for 24 hours in a dessicator at 1.33 kPa. The melting points were determined on a Boëtius apparatus HMK 73/4615 (VEB Analytik, Dresden, Germany) and are uncorrected. Elemental analyses were performed with an EA 1110 CHNS Analyzer (Carlo Erba). UV spectra (λ, nm) were determined on a Waters Photodiode Array Detector 2996 (Waters Corp., Milford, MA, USA) in *ca*. 6 × 10^−4^ M methanolic solution and log ε (the logarithm of molar absorption coefficient ε) was calculated for the absolute maximum λ_max_ of individual target compounds. Infrared spectra were recorded using KBr pellets on the FT-IR spectrometer Nicolet 6700 (Nicolet–Thermo Scientific, USA). Wavenumbers are given in cm^−1^. All ^1^H-NMR and ^13^C-NMR spectra were recorded with a Varian Mercury-VxBB 300 spectrometer (299.95 MHz for ^1^H and 75.43 MHz for ^13^C; Varian Corp., Palo Alto, CA, USA). Chemical shifts were recorded as δ values in ppm and were indirectly referenced to tetramethylsilane (TMS) via the solvent signal (2.49 for ^1^H, 39.7 for ^13^C in DMSO-*d*_6_).

### 3.2. Synthesis

#### 3.2.1. General procedure for synthesis of arylmethylidenerhodanines **1–16**

An equimolar amounts of an aldehyde and rhodanine or 3-(2-hydroxyethyl)rhodanine (0.015 mol) were heated under a reflux condenser with ethanol (15 mL) and concentrated ammonia solution (1.1 mL) until all solid components dissolved. The solution of ammonium chloride (1.00 g) in 2 mL of hot (80 °C) distilled water was then added, and the reaction mixture was refluxed for 2 hours. After cooling, the separated solid was filtered through a sintered glass, washed with distilled water (50 mL) and then with 50% ethanol (50 mL). The product was crystallized from anhydrous ethanol.

*(5Z)-5-Benzylidene-2-thioxo-1,3-thiazolidin-4-one* (**1**). Yellow crystalline compound; Yield 65%; Mp 206–207 °C (205 [26], 203–205 °C [27]); Anal. Calcd. for C_10_H_7_NOS_2_ (221.30): C 54.27%, H 3.19%, N 6.33%, S 28.98%; found: C 54.03%, H 3.13%, N 6.35%, S 26.42%; UV (nm), λ_max_/log ε: 376.0/3.31; IR (KBr, cm^−1^): 3154 (NH), 1700 (C=O); ^1^H-NMR (DMSO-*d_6_*), δ: 7.63 (1H, s, CH), 7.61–7.45 (5H, m, H2, H3, H4, H5, H6); ^13^C-NMR (DMSO-*d_6_*), δ: 195.9, 169.6, 133.2, 131.9, 131.0, 130.7, 129.7, 125.7.

*(5Z)-5-(2-Hydroxybenzylidene)-2-thioxo-1,3-thiazolidin-4-one* (**2a**). Yellow crystalline compound; Yield 57%; Mp 219–226 °C (224–225 °C [27]); Anal. Calcd. for C_10_H_7_NO_2_S_2_ (237.30): C 50.61%, H 2.97%, N 5.90%, S 27.03%; found: C 50.81%, H 2.94%, N 5.83%, S 26.93%; UV (nm), λ_max_/log ε: 394.1/3.38; IR (KBr, cm^−1^): 3153 (NH), 1700 (C=O); ^1^H-NMR (DMSO-*d_6_*), δ: 13.73 (1H, bs, NH), 10.66 (1H, bs, OH), 7.84 (1H, s, CH), 7.37–7.26 (2H, m, H4 and H6), 6.99–6.90 (2H, m, H3 and H5); ^13^C-NMR (DMSO-*d_6_*), δ: 196.2, 169.8, 157.8, 133.0, 129.5, 127.5, 124.0, 120.2, 120.1, 116.4.

*(5Z)-5-(3-Hydroxybenzylidene)-2-thioxo-1,3-thiazolidin-4-one* (**2b**). Yellow crystalline compound; Yield 64%; Mp 244–251 °C (237–239 °C [27]); Anal. Calcd. for C_10_H_7_NO_2_S_2_ (237.30): C 50.61%, H 2.97%, N 5.90%, S 27.03%; found: C 50.45%, H 2.80%, N 5.93%, S 25.39%; UV (nm), λ_max_/log ε: 365.0/3.38; IR (KBr, cm^−1^): 3343 (OH), 3169 (NH), 1699 (C=O); ^1^H-NMR (DMSO-*d_6_*), δ: 9.86 (1H, bs, OH), 7.53 (1H, s, CH), 7.32 (1H, t, *J* = 8.0, H5), 7.06–7.01 (1H, m, H6), 6.96 (1H, t, *J* = 1.9, H2), 6.91–6.86 (1H, m, H4); ^13^C-NMR (DMSO-*d_6_*), δ: 196.0, 169.6, 158.2, 134.3, 132.1, 130.8, 125.5, 122.1, 118.3, 116.4.

*(5Z)-5-(4-Hydroxybenzylidene)-2-thioxo-1,3-thiazolidin-4-one* (**2c**). Orange crystalline compound; Yield 78%; Mp 294–295 °C (279–280 °C [27]). Anal. Calcd. for C_10_H_7_NO_2_S_2_ (237.30): C 50.61%, H 2.97%, N 5.90%, S 27.03%; found: C 50.42%, H 2.91%, N 5.82%, S 25.94%; UV (nm), λ_max_/log ε: 392.9/3.35; IR (KBr, cm^−1^): 3393 (OH), 3145 (NH), 1688 (C=O); ^1^H-NMR (DMSO-*d_6_*), δ: 10.42 (1H, bs, OH), 7.55 (1H, s, CH), 7.50–7.41 (2H, m, AA´, BB´, H2 and H6), 6.95–6.87 (2H, m, AA´, BB´, H3 and H5); ^13^C-NMR (DMSO-*d_6_*), δ: 195.7, 169.7, 130.6, 133.3, 132.7, 124.2, 121.1, 116.8.

*(5Z)-5-(2,4-Hydroxybenzylidene)-2-thioxo-1,3-thiazolidin-4-one* (**3**). Orange crystalline compound; Yield 81%; Mp 272–276 °C (>300 [28]). Anal. Calcd. for C_10_H_7_NO_3_S_2_ (253.30): C 47.42%, H 2.79%, N 5.53%, S 25.32%; found: C 46.27%, H 3.40%, N 6.45%, S 24.03%; UV (nm), λ_max_/log ε: 407.3/3.41; IR (KBr, cm^−1^): 3197, 3139 (NH), 1683 (C=O); ^1^H-NMR (DMSO-*d_6_*), δ: 10.48 (2H, bs, OH), 7.73 (1H, s, CH), 7.13 (1H, d, *J* = 9.1 Hz, H6´), 6.44–6.34 (2H, m, H3´, H5´); ^13^C-NMR (DMSO-*d_6_*), δ: 197.2, 172.4, 162.1, 159.8, 131.1, 126.7, 120.9, 112.4, 108.9, 102.7.

*(5Z)-5-(2-Methoxybenzylidene)-2-thioxo-1,3-thiazolidin-4-one* (**4a**). Yellow crystalline compound; Yield 95%; Mp 257–258 °C (205–206 [28]). Anal. Calcd. for C_11_H_9_NO_2_S_2_ (251.32): C 52.57%, H 3.61%, N 5.57%, S 25.52%; found: C 52.87%, H 3.34%, N 5.67%, S 25.09%; UV (nm), λ_max_/log ε: 389.2/3.46; IR (KBr, cm^−1^): 3141(NH), 1705 (C=O); ^1^H-NMR (DMSO-*d_6_*), δ: 13.76 (1H, bs, NH), 7.78 (1H, s, CH), 7.53–7.44 (1H, m, H4´), 7.37 (1H, dd, *J* = 7.7 Hz, *J* = 1.7 Hz, H6´), 7.14 (1H, d, *J* = 7.7 Hz, H3´), 7.08 (1H, t, *J* = 7.7 Hz, H5´), 3.88 (3H, s, OCH_3_); ^13^C-NMR (DMSO-*d_6_*), δ: 196.3, 169.6, 158.3, 133.2, 129.9, 126.9, 125.5, 121.5, 121.4, 112.2, 56.0.

*(5Z)-5-(3-Methoxybenzylidene)-2-thioxo-1,3-thiazolidin-4-one* (**4b**). Yellow crystalline compound; Yield 81%; Mp 236–237 °C (232 °C [29]); Anal. Calcd. for C_11_H_9_NO_2_S_2_ (251.32): C 52.57%, H 3.61%, N 5.57%, S 25.52%; found: C 52.73%, H 5.43%, N 5.65%, S 25.87%; UV (nm), λ_max_/log ε: 379.6/3.41; IR (KBr, cm^−^^1^): 3151 (NH), 1698 (C=O); ^1^H-NMR (DMSO-*d_6_*), δ: 13.82 (1H, bs, NH), 7.60 (1H, s, CH), 7.44 (1H, t, *J* = 8.1 Hz, H5´), 7.16–7.03 (3H, m, H2´, H4´, H6´), 3.79 (3H, s, OCH_3_); ^13^C-NMR (DMSO-*d_6_*), δ: 195.8, 169.5, 159.9, 134.5, 131.8, 130.7, 126.0, 122.6, 116.9, 115.8, 55.5.

*(5Z)-5-(4-Methoxybenzylidene)-2-thioxo-1,3-thiazolidin-4-one* (**4c**). Yellow crystalline compound; Yield 90%; Mp 260–261 °C (261–262 °C [30]); Anal. Calcd. for C_11_H_9_NO_2_S_2_ (251.32): C 52.57%, H 3.61%, N 5.57%, S 25.52%; found: C 52.25%, H 3.60%, N 5.75%, S. 27.94%; UV (nm), λ_max_/log ε: 385.6/3.48; IR (KBr, cm^−^^1^): 3137 (NH), 1687 (C=O); ^1^H-NMR (DMSO-*d_6_*), δ: 13.72 (1H, bs, NH), 7.59 (1H, s, CH), 7.58–7.50 (2H, m, AA´, BB´, H2´, H6´), 7.13–7.05 (2H, m, AA´, BB´, H3´, H5´), 3.82 (3H, s, OCH_3_); ^13^C-NMR (DMSO-*d_6_*), δ: 195.7, 169.6, 161.5, 132.9, 132.1, 125.7, 122.4, 115.3, 55.8.

*(5Z)-5-(4-Hydroxy-3-methoxybenzylidene)-2-thioxo-1,3-thiazolidin-4-one* (**5**). Yellow crystalline compound; Yield 75%; Mp 233–234 °C (227–228 °C [31]); Anal. Calcd. for C_11_H_9_NO_3_S_2_ (267.32): C 49.42%, H 3.39%, N 5.24%, S 23.99%; found: C 49.46%, H 3.17%, N 5.24%, S 22.56%; UV (nm), λ_max_/log ε: 406.1/3.33; IR (KBr, cm^−1^): 3340 (OH), 3269 (NH), 1714 (C=O); ^1^H-NMR (DMSO-*d_6_*), δ: 10.09 (1H, bs, OH), 7.56 (1H, s, CH), 7.14 (1H, d, *J* = 2.1, H2), 7.07 (1H, dd, *J* = 8.4 and 2.1, H6), 6.92 (1H, d, *J* = 8.4, H5), 3.82 (3H, s, OCH_3_); ^13^C-NMR (DMSO-*d_6_*), δ: 195.7, 169.7, 150.2, 148.3, 133.0, 125.3, 124.6, 121.3, 116.6, 114.5, 55.8.

*(5Z)-5-[(4-Dimethylamino)benzylidene]-2-thioxo-1,3-thiazolidin-4-one* (**6**). Orange crystalline compound; Yield 90%; Mp 283–286 °C (283–284 °C [30]); Anal. Calcd. for C_12_H_12_N_2_OS_2_ (264.37): C 54.52%, H 4.58%, N 10.60%, S 24.26%; found: C 54.38%, H 4.75%, N 10.68%, S 22.70%; UV (nm), λ_max_/log ε: 463.0/3.39; IR (KBr, cm^−1^): 3138 (NH), 1683 (C=O); ^1^H-NMR (DMSO-*d_6_*), δ: 13.55 (1H, bs, NH), 7.49 (1H, s, CH), 7.45–7.36 (2H, m, AA´, BB´, H2´, H6´), 6.84–6.76 (2H, m, AA´, BB´, H3´, H5´), 3.01 (6H, s, NCH_3_); ^13^C-NMR (DMSO-*d_6_*), δ: 195.2, 169.6, 151.9, 133.5, 133.1, 120.0, 117.5, 112.4, 39.8.

*(5Z)-5-(2-Nitrobenzylidene)-2-thioxo-1,3-thiazolidin-4-one* (**7a**). Yellow crystalline compound; Yield 68%; Mp 190–195 °C (204–205 °C [32]); Anal. Calcd. for C_10_H_6_N_2_O_3_S_2_ (266.30): C 45.10%, H 2.27%, N 10.52%, S 24.08%; found: C 43.78%, H 1.53%, N 10.14%, S 26.31%; UV (nm), λ_max_/log ε: 360.1/3.43; IR (KBr, cm^−1^): 3098 (NH), 1735 (C=O); ^1^H-NMR (DMSO-*d_6_*), δ: 13.93 (1H, bs, NH), 8.19 (1H, d, *J* = 8.2 Hz, H3´), 7.92–7.84 (1H, m, H5´), 7.86 (1H, s, CH), 7.76–7.66 (2H, m, H4´, H6´); ^13^C-NMR (DMSO-*d_6_*), δ: 196.0, 168.8, 148.1, 134.8, 131.5, 130.5, 129.6, 129.0, 128.1, 125.8.

*(5Z)-5-(3-Nitrobenzylidene)-2-thioxo-1,3-thiazolidin-4-one* (**7b**). Yellow crystalline compound; Yield 71%; Mp 257–263 °C (263–264 °C [33]); Anal. Calcd. for C_10_H_6_N_2_O_3_S_2 _(266.30): C 45.10%, H 2.27%, N 10.52%, S 24.08%; found: C 44.64%, H 2.41%, N 11.07%, S 24.32%; UV (nm), λ_max_/log ε: 367.6/3.38; IR (KBr, cm^−1^): 3255, 3182 (NH), 1728 (C=O); ^1^H-NMR (DMSO-*d_6_*), δ: 8.39 (1H, t, *J* = 2.0 Hz, H2´), 8.26 (1H, ddd, *J* = 8.1 Hz, *J* = 2.0 Hz, *J* = 0.8 Hz, H4´), 7.96 (1H, d, *J* = 8.1 Hz, H6´), 7.79 (1H, t, *J* = 8.1 Hz, H5´), 7.70 (1H, s, CH); ^13^C-NMR (DMSO-*d_6_*), δ: 196.2, 171.2, 148.5, 135.9, 135.2, 131.1, 130.1, 128.1, 124.8, 124.6.

*(5Z)-5-(4-Nitrobenzylidene)-2-thioxo-1,3-thiazolidin-4-one* (**7c**). Orange crystalline compound; Yield 58%; Mp 272–276 °C (273–274 °C [32]); Anal. Calcd. for C_10_H_6_N_2_O_3_S_2_ (266.30): C 45.10%, H 2.27%, N 10.52%, S 24.08%; found: C 45.15%, H 2.28%, N 10.52%, S 24.31%; UV (nm), λ_max_/log ε: 394.1/3.37; IR (KBr, cm^−1^): 3274, 3105 (NH), 1728 (C=O); ^1^H-NMR (DMSO-*d_6_*), δ: 8.35–8.26 (2H, m, AA´, BB´, H3´, H5´), 7.86–7.77 (2H, m, AA´, BB´, H2´, H6´), 7.70 (1H, s, CH); ^13^C-NMR (DMSO-*d_6_*), δ: 195.5, 169.5, 147.7, 139.4, 131.5, 130.1, 128.8, 124.5.

*(5Z)-5-(2-Fluorobenzylidene)-2-thioxo-1,3-thiazolidin-4-one* (**8a**). Yellow crystalline compound; Yield 67%; Mp 201–203 °C (201–203 °C [27]). Anal. Calcd. for C_10_H_6_FNOS_2_ (239.29): C 50.19%, H 2.53%, N 5.85%, S 26.80%; found: C 50.27%, H 2.70%, N 6.11%, S 26.42%; UV (nm), λ_max_/log ε: 370.0/3.48; IR (KBr, cm^−1^): 3159 (NH), 1698 (C=O); ^1^H-NMR (DMSO-*d_6_*), δ: 13.91 (1H, bs, NH), 7.59 (1H, s, CH), 7.58–7.32 (4H, m, H3´, H4´, H5´, H6´); ^13^C-NMR (DMSO-*d_6_*), δ: 195.6, 169.4, 160.8 (d, *J* = 252.3 Hz), 133.3 (d, *J* = 8.6 Hz), 129.6, 128.3, 125.8 (d, *J* = 3.5 Hz), 122.5 (d, *J* = 6.3 Hz), 121.1 (d, *J* = 11.5 Hz), 116.5 (d, *J* = 21.3 Hz).

*(5Z)-5-(3-Fluorobenzylidene)-2-thioxo-1,3-thiazolidin-4-one* (**8b**). Yellow crystalline compound; Yield 46%; Mp 199–200 °C (201–202 °C [27]). Anal. Calcd. for C_10_H_6_FNOS_2_ (239.29): C 50.19%, H 2.53%, N 5.85%, S 26.80%; found: C 50.36%, H 2.72%, N 6.05%, S 26.31%; UV (nm), λ_max_/log ε: 384.2/3.47; IR (KBr, cm^−1^): 3184 (NH), 1705 (C=O); ^1^H-NMR (DMSO-*d_6_*), δ: 13.88 (1H, bs, NH), 7.63 (1H, s, CH), 7.62–7.52 (1H, m, H6´), 7.48–7.29 (3H, m, H2´, H4´, H5´); ^13^C-NMR (DMSO-*d_6_*), δ: 195.6, 169.5, 162.5 (d, *J* = 245.3 Hz), 135.5 (d, *J* = 8.1 Hz), 131.7 (d, *J* = 8.7 Hz), 130.2 (d, *J* = 2.3 Hz), 127.4, 126.1 (d, *J* = 8.9 Hz), 117.7 (d, *J* = 21.4 Hz), 117.3 (d, *J* = 22.5 Hz).

*(5Z)-5-(4-Fluorobenzylidene)-2-thioxo-1,3-thiazolidin-4-one* (**8c**). Yellow crystalline compound; Yield 78%; Mp 225–227 °C (226–227 °C [27]). Anal. Calcd. for C_10_H_6_FNOS_2_ (239.29): C 50.19%, H 2.53%, N 5.85%, S 26.80%; found: C 50.00%, H 2.51%, N 5.87%, S 26.74%; UV (nm), λ_max_/log ε: 386.7/3.45; IR (KBr, cm^−^^1^): 3103 (NH), 1724 (C=O); ^1^H-NMR (DMSO-*d_6_*), δ: 13.83 (1H, bs, NH), 7.70–7.60 (2H, m, H2´, H6´), 7.64 (1H, s overlapped, CH), 7.58-7.32 (2H, m, H3´. H5´); ^13^C-NMR (DMSO-*d_6_*), δ: 195.8, 169.6, 163.2 (d, *J* = 251.7 Hz), 133.2 (d, *J* = 8.7 Hz), 130.7, 129.9 (d, *J* = 3.4 Hz), 125.4 (d, *J* = 2.9 Hz), 116.8 (d, *J* = 21.9 Hz).

*(5Z)-5-(2-Chlorobenzylidene)-2-thioxo-1,3-thiazolidin-4-one* (**9a**). Yellow crystalline compound; Yield 48%; Mp 191–193 °C (192 °C [34]); Anal. Calcd. for C_10_H_6_ClNOS_2_ (255.74): C 46.96%, H 2.36%, N 5.48%, S 25.08%; found: C 47.06%, H 2.38%, N 5.41%, S 25.49%; UV (nm), λ_max_/log ε: 365.0/3.29; IR (KBr, cm^−1^): 3069 (NH), 1734, 1698 (C=O); ^1^H-NMR (DMSO-*d_6_*), δ: 13.93 (1H, bs, NH), 7.74 (1H, s, CH), 7.66–7.60 (1H, m, H3´), 7.54–7.47 (3H, m, H4´, H5´, H6´); ^13^C-NMR (DMSO-*d_6_*), δ: 195.7, 169.3, 135.0, 132.3, 131.0, 130.7, 129.5, 129.3, 128.5, 126.3.

*(5Z)-5-(3-Chlorobenzylidene)-2-thioxo-1,3-thiazolidin-4-one* (**9b**). Orange crystalline compound; Yield 76%; Mp 234–235 °C (233 °C [34]); Anal. Calcd. for C_10_H_6_ClNOS_2_ (255.74): C 46.96%, H 2.36%, N 5.48%, S 25.08%; found: C 46.86%, H 2.28%, N 5.48%, S 25.71%; UV (nm), λ_max_/log ε: 376.2/3.31; IR (KBr, cm^−1^): 3109 (NH), 1718 (C=O); ^1^H-NMR (DMSO-*d_6_*), δ: 13.90 (1H, bs, NH), 7.68 (1H, s, CH), 7.62–7.47 (4H, m, H2´, H4´, H5´, H6´); ^13^C-NMR (DMSO-*d_6_*), δ: 195.5, 169.4, 135.3, 134.2, 131.4, 130.5, 130.4, 130.0, 128.3, 127.5.

*(5Z)-5-(4-Chlorobenzylidene)-2-thioxo-1,3-thiazolidin-4-one* (**9c**). Yellow crystalline compound; Yield 88%; Mp 232–233 °C (230–231 °C [33]); Anal. Calcd. for C_10_H_6_ClNOS_2_ (255.74): C 46.96%, H, 2.36%, N 5.48%, S 25.08%; found: C 46.87%, H 2.78%, N 5.60%, S 24.02%. UV (nm), λ_max_/log ε: 379.6/3.36; IR (KBr, cm^−1^): 3150 (NH), 1709 (C=O). ^1^H-NMR (DMSO-*d_6_*), δ: 13.87 (1H, bs, NH), 7.62 (1H, s, CH), 7.60–7.58 (4H, m, H2´, H3´, H5´, H6´); ^13^C-NMR (DMSO-*d_6_*), δ: 195.6, 169.5, 135.6, 132.3, 132.1, 130.4, 129.7, 126.5.

*(5Z)-5-(2-Bromobenzylidene)-2-thioxo-1,3-thiazolidin-4-one* (**10a**). Yellow crystalline compound; Yield 59%; Mp 185–186 °C (183.5 °C [34]); Anal. Calcd. for C_10_H_6_BrNOS_2_ (300.19): C 40.01%, H, 2.01%, N 4.67%, S 21.36%; found: C 39.90%, H 1.89%, N 4.62%, S 22.01%; UV (nm), λ_max_/log ε: 365.0/3.46; IR (KBr, cm^−1^): 3150 (NH), 1709 (C=O). ^1^H-NMR (DMSO-*d_6_*), δ: 13.97 (1H, bs, NH), 7.82–7.78 (1H, m, H3´), 7.70 (1H, s, CH), 7.61–7.47 (2H, m, H4´, H6´), 7.45–7.37 (1H, m, H5´); ^13^C-NMR (DMSO-*d_6_*), δ: 195.8, 169.3, 133.9, 132.7, 132.4, 129.6, 129.3, 129.1, 129.0, 125.9.

*(5Z)-5-(3-Bromobenzylidene)-2-thioxo-1,3-thiazolidin-4-one* (**10b**). Yellow crystalline compound; Yield 45%; Mp 244–246 °C (238 °C [34]); Anal. Calcd. for C_10_H_6_BrNOS_2_ (300.19): C 40.01%, H 2.01%, N 4.67%, S 21.36%; found: C 40.14%, H 1.98%, N 4.53%, S 22.19%; UV (nm), λ_max_/log ε: 379.9/3.46; IR (KBr, cm^−1^): 3111 (NH), 1717 (C=O). ^1^H-NMR (DMSO-*d_6_*), δ: 13.88 (1H, bs, NH), 7.80 (1H, s, H2´), 7.71–7.64 (1H, m, H4´), 7.61 (1H, s, CH), 7.55 (1H, d, *J* = 7.6 Hz, H6´), 7.48 (1H, t, *J* = 7.6 Hz, H5´); ^13^C-NMR (DMSO-*d_6_*), δ: 195.6, 169.5, 135.6, 133.4, 133.3, 131.6, 129.9, 128.7, 127.5, 122.7.

*(5Z)-5-(4-Bromobenzylidene)-2-thioxo-1,3-thiazolidin-4-one* (**10c**). Yellow crystalline compound; Yield 92%; Mp 236–238 °C (238–239 °C [27]); Anal. Calcd. for C_10_H_6_BrNOS_2_ (300.19): C 40.01%, H 2.01%, N 4.67%, S 21.36%; found: C 39.95%, H 1.87%, N 4.64%, S 21.78%; UV (nm), λ_max_/log ε: 382.0/3.39; IR (KBr, cm^−1^): 3150 (NH); 1708 (C=O); ^1^H-NMR (DMSO-*d_6_*), δ: 7.76–7.69 (2H, m, AA´, BB´, H2´, H6´), 7.60 (1H, s, CH), 7.55–7.49 (2H, m, AA´, BB´, H3´, H5´); ^13^C-NMR (DMSO-*d_6_*), δ: 195.6, 169.5, 132.6, 132.4, 132.4, 130.5, 126.5, 124.5.

*(5Z)-3-(2-Hydroxyethyl)-5-(2-nitrobenzylidene)-2-thioxo-1,3-thiazolidin-4-one* (**11a**). The product was separated from the reaction mixture by means of column chromatography using Silicagel 60 Fluka (0.040–0.063 mm) as adsorbent and light petroleum/ethyl acetate 6:4 as mobile phase. After crystallization from ethanol a yellow crystalline compound was obtained. Yield 5%; Mp 105–107 °C; Anal. Calcd. for C_10_H_6_BrNOS_2_ (310.35): C 46.44%, H 3.25%, N 9.03%, S 20.66%; found: C 46.60%, H 3.25%, N 8.96%, S 20.23%; UV (nm), λ_max_/log ε: 361.0/3.46; IR (KBr, cm^−1^): 3458 (OH), 1716 (C=O); ^1^H-NMR (DMSO-*d_6_*), δ: 8.39–8.26 (2H, m, AA´, BB´, H3´, H5´), 7.91–7.84 (2H, m, AA´, BB´, H2´, H6´), 7.88 (1H, s, overlapped, CH), 4.94 (1H, bs, OH), 4.11 (2H, t, *J* = 5.9 Hz, NCH_2_), 3.73–3.59 (2H, m, OCH_2_); ^13^C-NMR (DMSO-*d_6_*), δ: 193.6, 167.2, 147.8, 139.3, 131.7, 129.8, 127.1, 124.6, 56.9, 46.9.

*(5Z)-3-(2-Hydroxyethyl)-5-(3-nitrobenzylidene)-2-thioxo-1,3-thiazolidin-4-one* (**11b**). The product was separated from the reaction mixture by means of column chromatography using Silicagel 60 Fluka (0.040–0.063 mm) as adsorbent and light petroleum/ethyl acetate 6:4 as mobile phase. After crystallization from ethanol a yellow crystalline compound was obtained. Yield 14%; Mp 217–220 °C; Anal. Calcd. for C_10_H_6_BrNOS_2_ (310.35): C 46.44%, H, 3.25%, N 9.03%, S 20.66%; found: C 46.79%, H 2.97%, N 9.12%, S 20.46%; UV (nm), λ_max_/log ε: 366.4/3.38; IR (KBr) 3448 (OH); 1716 (C=O); ^1^H-NMR (DMSO-*d_6_*), δ: 8.47 (1H, t, *J* = 1.9 Hz, H2´), 8.33–8.27 (1H, m, H4´), 8.02 (1H, d, *J* = 8.0 Hz, H6´), 7.94 (1H, s, CH), 7.82 (1H, t, *J* = 8.0 Hz, H5´), 4.93 (1H, t, *J* = 6.0 Hz, OH), 4.12 (2H, t, *J* = 6.0 Hz, NCH_2_), 3.65 (2H, q, *J* = 6.0 Hz, OCH_2_); ^13^C-NMR (DMSO-*d_6_*), δ: 193.4, 167.1, 148.5, 135.9, 134.8, 131.3, 130.2, 125.7, 125.2, 125.0, 56.9, 46.9.

*(5Z)-3-(2-Hydroxyethyl)-5-(4-nitrobenzylidene)-2-thioxo-1,3-thiazolidin-4-one* (**11c**). The product was separated from the reaction mixture by means of column chromatography using Silicagel 60 Fluka (0.040–0.063 mm) as adsorbent and light petroleum/ethyl acetate 6:4 as mobile phase. After crystallization from ethanol a red crystalline compound was obtained. Yield 16%; Mp 202–205 °C (204–205 °C [24]); Anal. Calcd. for C_10_H_6_BrNOS_2_ (310.35): C 46.44%, H 3.25%, N 9.03%, S 20.66%; found: C 46.66%, H 3.37%, N 8.87%, S 20.15%; UV (nm), λ_max_/log ε: 386.8/3.39; IR (KBr, cm^−1^): 3421 (OH), 1713 (C=O); ^1^H-NMR (DMSO-*d_6_*), δ: 8.39–8.26 (2H, m, AA´, BB´, H3´, H5´), 7.91–7.84 (2H, m, AA´, BB´, H2´, H6´), 7.88 (1H, s, overlapped, CH), 4.94 (1H, bs, OH), 4.11 (2H, t, *J* = 5.9 Hz, NCH_2_), 3.73–3.59 (2H, m, OCH_2_); ^13^C-NMR (DMSO-*d_6_*), δ: 193.6, 167.2, 147.8, 139.3, 131.7, 129.8, 127.1, 124.6, 56.9, 46.9.

*(5Z)-5-(Pyridin-2-ylmethylidene)-2-thioxo-1,3-thiazolidin-4-one* (**12**). Yellow crystalline compound; Yield 70%; Mp 269–272 °C (268 °C [26]); Anal. Calcd. for C_9_H_6_N_2_OS_2_ (222.29): C 48.63%, H 2.72%, N 12.60%, S 28.85%; found: C 48.26%, H 2.65%, N 12.82%, S 28.90%; UV (nm), λ_max_/log ε: 349.9/3.44; IR (KBr, cm^−1^): 3096 (NH), 1726 (C=O); ^1^H-NMR (DMSO-*d_6_*), δ: 13.66 (1H, bs, NH), 8.77 (1H, d, *J* = 4.7 Hz, H6´), 7.94 (1H, dt, *J* = 7.6 Hz, *J* = 1.8 Hz, H4´), 7.88 (1H, d, *J* = 7.6 Hz, H3´), 7.67 (1H, s, CH), 7.45–7.39 (1H, m, H5´); ^13^C-NMR (DMSO-*d_6_*), δ: 202.2, 169.5, 151.3, 149.7, 137.8, 129.9, 128.3, 127.6, 124.2.

*(5Z)-5-(Pyridin-3-ylmethylidene)-2-thioxo-1,3-thiazolidin-4-one* (**13**). Yellow crystalline compound; Yield 68%; Mp 309–313 °C (295 °C [26]); Anal. Calcd. for C_9_H_6_N_2_OS_2_ (222.29): C 48.63%, H 2.72%, N 12.60%, S 28.85%; found: C 48.49%, H 2.81%, N 12.41%, S 28.45%; UV (nm), λ_max_/log ε: 356.7/3.46; IR (KBr, cm^−1^): 3431 (NH), 1709 (C=O); ^ 1^H-NMR (DMSO-*d_6_*), δ: 8.82 (1H, d, *J* = 1.9 Hz, H2), 8.62 (1H, dd, *J* = 4.8 Hz, *J* = 1.9 Hz, H6), 7.96–7.88 (1H, m, H4), 7.66 (1H, s, CH), 8.62 (1H, dd, *J* = 4.8 Hz, *J* = 1.9 Hz, H5); ^13^C-NMR (DMSO-*d_6_*), δ: 195.5, 169.4, 151.9, 150.9, 136.5, 129.3, 128.3, 128.0, 124.5.

*(5Z)-5-(Pyridin-4-ylmethylidene)-2-thioxo-1,3-thiazolidin-4-one* (**14**). Orange crystalline compound; Yield 87%; Mp 320–322 °C (295 °C [26]); Anal. Calcd. for C_9_H_6_N_2_OS_2_ (222.29): C 48.63%, H 2.72%, N 12.60%, S 28.85%; found: C 48.53%, H 3.22%, N 12.68%, S 29.12%; UV (nm), λ_max_/log ε: 358.1/3.46; IR (KBr, cm^−1^): 3420 (NH), 1701 (C=O); ^1^H-NMR (DMSO-*d_6_*), δ: 8.74–8.68 (2H, m, H2´, H6´), 7.55 (1H, s, CH), 7.54–7.50 (2H, m, H3´, H5´); the ^13^C-NMR spectrum could not been recorded due to the poor solubility of the compound.

*(5Z)-5-(Pyrazin-2-ylmethylidene)-2-thioxo-1,3-thiazolidin-4-one* (**15**). Orange crystalline compound; Yield 57%; Mp 310 °C; Anal. Calcd. for C_8_H_5_N_3_OS_2_ (223.27): C 43.03%, H 2.26%, N 18.82%, S 28.72%; found: C 43.12%, H 1.96%, N 18.72%, S 28.29%; UV (nm), λ_max_/log ε: 374.8/3.39; IR (KBr, cm^−1^): 3198 (NH), 1715, 1704 (C=O); ^1^H-NMR (DMSO-*d_6_*), δ: 13.78 (1H, bs, NH), 9.09 (1H, d, *J* = 1.4 Hz, H3´), 8.85–8.80 (1H, m, H5´), 8.63 (1H, d, *J* = 2.7 Hz, H6´), 7.73 (1H, s, CH); ^13^C-NMR (DMSO-*d_6_*), δ: 200.9, 169.3, 148.6, 147.4, 144.6, 144.4, 132.2, 124.2.

*(5Z)-5-(Furan-2-ylmethylidene)-2-thioxo-1,3-thiazolidin-4-one* (**16**). Orange crystalline compound; Yield 55%; Mp 235–237 °C (230–231°C [33]); Anal. Calcd. for C_8_H_5_NO_2_S_2_ (211.26): C 45.48%, H 2.39%, N 6.63%, S 30.36%; found: C 45.44%, H 2.48%, N 6.43%, S 30.44%; UV (nm), λ_max_/log ε: 395.1/3.29; IR (KBr, cm^−1^): 3141 (NH), 1689 (C=O); ^1^H-NMR (DMSO-*d_6_*), δ: 13.67 (1H, bs, NH), 8.09 (1H, dd, *J* = 1.9 Hz, *J* = 0.69 Hz, H5), 7.47 (1H, s, CH), 7.16 (1H, dd, *J* = 3.6 Hz, *J* = 0.6 Hz, H3), 6.75 (1H, dd, *J* = 3.6 Hz, *J* = 1.9 Hz, H4); ^13^C-NMR (DMSO-*d_6_*), δ: 196.7, 169.2, 149.6, 148.5, 122.6, 120.1, 117.9, 114.1.

### 3.2. Lipophilicity HPLC Determination (capacity factor k/calculated log k)

A Waters Alliance 2695 XE HPLC separation module, a Waters Photodiode Array Detector Waters Alliance 2695 XE HPLC separation module and a Waters Photodiode Array Detector 2996 (Waters Corp., Milford, MA, USA) were used. Waters Symmetry^®^ C_18_ 5 μm, 4.6 × 250 mm, Part No. WAT054275 (Waters Corp., Milford, MA, USA) chromatographic column was used. The HPLC separation process was monitored by Empower™ 2 Chromatography Data Software, Waters 2009 (Waters Corp., Milford, MA, USA). The mixture of MeOH (HPLC grade, 70%) and H_2_O (HPLC – Mili-Q Grade, 30%) was used as a mobile phase. The total flow rate of the column was 0.9 mL/min; injection volume 30 μL, column temperature 30 °C and sample temperature 10 °C were used. The detection wavelength of 210 nm was chosen. The KI methanolic solution was used for the dead time (t_D_) determination. Retention times (t_R_) were measured in minutes. The capacity factors *k* were calculated using the Empower™ 2 Chromatography Data Software according to formula *k* = (t_R_ − t_D_)/t_D_, where t_R_ is the retention time of the solute, whereas t_D_ denotes the dead time obtained using an unretained analyte. Log *k*, calculated from the capacity factor *k*, is used as the lipophilicity index converted to log *P* scale. The log *k* values of the individual compounds are shown in Table 1.

### 3.3. Study of Photosynthetic Electron Transport (PET) Inhibition in Spinach Chloroplasts

Chloroplasts were prepared from spinach (*Spinacia oleracea* L.) according to ref. [54]. The inhibition of photosynthetic electron transport (PET) in spinach chloroplasts was determined spectrophotometrically (Genesys 6, Thermo Scientific, USA) using an artificial electron acceptor 2,6-dichlorophenol-indophenol (DCIPP) according to ref. [55], and the rate of photosynthetic electron transport was monitored as a photoreduction of DCPIP. The measurements were carried out in phosphate buffer (0.02 mol/L, pH 7.2) containing sucrose (0.4 mol/L), MgCl_2_ (0.005 mol/L) and NaCl (0.015 mol/L). The chlorophyll content was 30 mg/L in these experiments, and the samples were irradiated (~100 W/m^2^) from 10 cm distance with a halogen lamp (250 W) using a 4 cm water filter to prevent warming of the samples (suspension temperature 22 °C). The studied compounds were dissolved in DMSO due to their limited water solubility. The applied DMSO concentration (up to 4%) did not affect the photochemical activity in spinach chloroplasts. The inhibitory efficiency of the studied compounds was expressed by IC_50_ values, *i.e.*, by molar concentration of the compounds causing 50% decrease in the oxygen evolution rate relative to the untreated control. The comparable IC_50_ value for the selective herbicide 3-(3,4-dichlorophenyl)-1,1-dimethylurea, DCMU (Diuron^®^) was about 1.9 μmol/L [56]. The results are summarized in Table 2.

### 3.4. Reduction of Chlorophyll Content in Green Algae *Chlorella vulgaris* Beij.

Green algae *Chlorella vulgaris* Beij. were cultivated statically at room temperature according to ref. [57] (photoperiod 16 h light/8 h dark; photosynthetically active radiation (PAR) 80 μmol/m^2^s; pH 7.2). The effect of rhodanine compounds on algal chlorophyll (Chl) content was determined after 7-day cultivation in the presence of the compounds tested, expressing the response as percentage of the corresponding values obtained for control. The Chl content in the algal suspension was determined spectrophotometrically (Genesys 6, Thermo Scientific, USA) after extraction into methanol according to Wellburn [58]. The Chl content in the suspensions at the beginning of cultivation was 0.01 mg/L. Because of their low water solubility, the tested compounds were dissolved in DMSO. DMSO concentration in the algal suspensions did not exceed 0.25% and at the end the control samples contained the same DMSO amount as the suspensions treated with the tested compounds. The antialgal activity of most effective compounds was expressed as IC_50_ value (the concentration of the inhibitor causing a 50% decrease in the content of Chl as compared with the control sample) or by percentual reduction of chlorophyll content (with respect to the control) after treatment with equimolar concentration of the studied compounds (100 μmol/L). The IC_50_ value for the standard, the selective herbicide 3-(3,4-dichlorophenyl)-1,1-dimethylurea, DCMU (Diuron^®^) was about 7.3 µmol/L. The results are summarized in Table 2.

## 4. Conclusions

The series of thirty rhodanine derivatives is presented. Their lipophilicity was determined using a well established RP-HPLC method. The compounds were tested for their activity related to inhibition of photosynthetic electron transport (PET) in spinach (*Spinacia oleracea* L.) chloroplasts and reduction of chlorophyll content in freshwater alga *Chlorella vulgaris*. Structure-activity relationships between the chemical structure, physical properties and biological activities of the evaluated compounds are discussed. (5*Z*)-5-(4-Bromobenzylidene)-2-thioxo-1,3-thiazolidin-4-one (**10c**) showed the highest PET-inhibition activity among the discussed compounds. (5*Z*)-5-(4-Chlorobenzylidene)-2-thioxo-1,3-thiazolidin-4-one (**9c**) expressed the highest reduction of chlorophyll content in freshwater alga *Chlorella vulgaris*. The results of the present study confirm previous observations regarding the influence of rhodanine and its derivatives on plants and show that both lipophilicity and character of substituents are important for their potency. These noteworthy compounds surely deserve further attention as potential pesticides and/or drugs.
